# Effectiveness of behavioral change techniques employed in eHealth interventions designed to improve glycemic control in persons with poorly controlled type 2 diabetes: a systematic review and meta-analysis protocol

**DOI:** 10.1186/s13643-017-0609-1

**Published:** 2017-10-24

**Authors:** Mihiretu Kebede, Lara Christianson, Zohaib Khan, Thomas L. Heise, Claudia R. Pischke

**Affiliations:** 1 University of Bremen, Health Sciences, Grazer Strasse 2, D-28359 Bremen, Germany; 2 Leibniz Institute for Prevention Research and Epidemiology – BIPS, Bremen, Germany; 3 University of Gondar, College of Medicine and Health Science, Institute of Public Health, Gondar, Ethiopia; 4 Khyber Medical University, Peshawar, Pakistan

**Keywords:** Systematic review protocol, Poorly controlled type 2 diabetes mellitus, Self-management, Interventions, eHealth, Glycemic control, Glycated hemoglobin, HbA1c

## Abstract

**Background:**

The incorporation of Behavioral Change Techniques (BCTs) in eHealth interventions for the management of non-communicable diseases (NCDs), such as type 2 diabetes mellitus (T2DM), might be a promising approach to improve clinical and behavioral outcomes of NCDs in the long run. This 3paper reports a protocol for a systematic review that aims to (a) identify the effects of individual BCTs in eHealth interventions for lowering glycated hemoglobin levels (HbA1c) and (b) investigate which additional intervention features (duration of intervention, tailoring, theory-base, and mode of delivery) affect levels of HbA1c in this population. The protocol follows the Preferred Reporting Items for Systematic review and Meta-Analysis Protocols (PRISMA-P) 2015 guideline.

**Methods/design:**

To identify eligible studies, an extensive systematic database search (PubMed, Web of Science, and PsycINFO) using keywords will be conducted. This review will include randomized controlled trials examining the effects of eHealth interventions on HbA1c in persons with poorly controlled T2DM over a minimum follow-up period of 3 months. Relevant data will be extracted from the included studies using Microsoft Excel. The content of the interventions will be extracted from the description of interventions and will be classified according to the BCT taxonomy v1 tool. The quality of studies will be independently assessed by two reviewers using the Cochrane risk of bias tool. If the studies have adequate homogeneity, meta-analysis will be considered. The effect sizes of each BCT will be calculated using the random effect model. The quality of the synthesized evidence will be evaluated employing the Grading of the Recommendations Assessment, Development and Evaluation (GRADE) criteria.

**Discussion:**

This systematic review is one of the firsts to appraise the effectiveness of eHealth interventions employing BCTs which aimed at improving glycemic control in persons with poorly controlled T2DM. The review will aggregate the effect sizes of BCTs on HbA1c levels. The results may inform future eHealth interventions targeting poorly controlled T2DM populations.

**Systematic review registration:**

PROSPERO CRD42016049940

**Electronic supplementary material:**

The online version of this article (10.1186/s13643-017-0609-1) contains supplementary material, which is available to authorized users.

## Background

Type 2 diabetes mellitus (T2DM) is a chronic metabolic disorder characterized by an inability to produce and/or utilize adequate amounts of the hormone insulin. This results in an inadequate metabolism of glucose leading to hyperglycemic conditions in the blood circulatory system [1]. Consistently, high blood sugar levels contribute to an increased risk for developing serious complications affecting the heart and blood vessels, eyes, kidneys, nerves, and teeth [1, 2]. Poor glycemic control in persons with diagnosed T2DM is one of the major challenges to effective diabetes management [3, 4]. Patients having a glycated hemoglobin (HbA1c) level of ≥ 7.0% are classified as having poorly controlled T2DM [5]. Patients suffering from poorly controlled T2DM have a higher risk of diabetic-related complications and mortality as well as lower quality of life [6, 7]. Effective diabetes management, especially the management of poor glycemic control, requires continuous monitoring of blood glucose levels, strict adherence to glucose lowering treatment regimens and lifestyle recommendations regarding diet and physical activity [1, 4]. According to the American Diabetes Association (2016), persons with T2DM are required to monitor their blood glucose levels frequently (e.g., fasting, before/after meals) [8, 9], to engage in at least 150 min/week of moderate aerobic physical activity spread over 2 to 3 days, avoiding the consumption of sugary beverages and low-fat or non-fat products with refined grains and added sugars is recommended [10].

Electronic Health (eHealth) interventions provide a successful mechanism for engaging patients with T2DM under chronic care [11, 12]. They are integral in supporting patients with T2DM in engaging in necessary self-management behaviors, such as regular blood glucose monitoring and insulin administration. Furthermore, behavioral counseling and information to increase knowledge regarding the risks and consequences of the disease can be effectively delivered to patients via eHealth interventions [13–17]. In general, participation in eHealth interventions that combine behavioral counseling and health education is associated with improvements in disease-related clinical and behavioral outcomes [12, 18]. However, there is a lack of research identifying the effects of the active ingredients of these multi-component interventions and their impact on changes in HbA1c levels in persons with poorly controlled T2DM.

The behavioral change technique (BCT) taxonomy is a comprehensive tool which helps researchers identify the active ingredients of behavioral interventions [19]. It contains 93 techniques to change behavior that are hierarchically clustered into 16 groups. This tool can be retrospectively applied to evaluate the contents of interventions outlined in the published literature. Thus far, two systematic reviews examined the effects of different BCTs employed in eHealth interventions targeting persons with poorly controlled T2DM [20, 21]. One systematic review analyzed the results of 13 RCTs and reported that BCTs, such as “feedback on performance” (for example, feedback on progress, feedback on action planning, feedback on quizzes, and feedback on personalized goal setting), providing information on consequences of behavior, problem-solving, and self-monitoring of behavior were associated with improvements in psychosocial well-being in persons with T2DM (i.e., reductions in depression and psychological distress) [22]. In a second review, Avery and colleagues (2015) identified successful strategies for the modification of physical activity and HbA1c-levels in this patient population. Two BCTs, namely “prompting the review of behavioral goals” and “providing information on where and when to perform physical activity” were reported as effective in reducing the mean HbA1c levels in persons with T2DM [21].

However, the results of our previous scoping review identified that no systematic review is currently available to investigate which BCTs are effective in lowering HbA1c levels of persons with poorly controlled T2DM [23]. Therefore, this review aims to identify effective BCTs and where possible to aggregate the effect sizes of BCTs across studies.

## Objectives

This review will systematically identify and evaluate the effectiveness of BCTs employed in eHealth interventions aimed at improving glycemic control in persons with poorly controlled T2DM.

The objectives of this systematic review are the following:

1. To investigate the effect of individual (or combined) BCTs employed in eHealth interventions targeting persons with poorly controlled T2DM on HbA1c-levels and
2. To examine which additional intervention features (duration of intervention, tailoring, theory-base, and mode of delivery) may affect levels of HbA1c in persons with poorly controlled T2DM

## Methods and analysis

This protocol follows the PRISMA-P (Preferred Reporting Items for Systematic review and Meta-Analysis Protocols) 2015 guideline [24] (see Additional file 1).

### Eligibility criteria

#### Study design

This systematic review will identify the contents of the interventions based on the latest version of BCT taxonomy [19]. In this review, only randomized controlled trials (RCTs) published between 1990 and June 2017 with a minimum follow-up period of 3 months that investigated the effects of eHealth interventions on patient populations with poorly controlled T2DM will be included.

Furthermore, studies will be included if

- Effects of eHealth interventions for the promotion of behavioral change among poorly controlled type 2 diabetes populations are reported.
- A change in mean HbA1c is reported as an outcome in both intervention and control groups.
- The results of the studies are published in English or in German

Articles not published in English or in German will be excluded from the review, as well as commentaries, short reports of interventions, letters to the editor, and editorials. In addition, eHealth interventions with the aim of improving knowledge and skills of health care providers related to the management of the disease will be excluded.

### Study populations

We will include studies with participants that are diagnosed with type 2 diabetes and over the age of 18 years and who have reported glycated hemoglobin (HbA1c) levels of > 7.0%.

### Interventions

We will analyze all eHealth-related interventions including mHealth (mobile Health), or other computer or mobile interventions (delivered via personal digital assistant (PDA), tablet, smartphone, games, web-based, apps), and all forms of information and communication technology (ICT)-based behavioral change interventions targeting persons with poorly controlled T2DM. This will include tailored or untailored interventions that aim to improve blood glucose monitoring by enhancing behavioral change for better self-management, physical activity, adherence to medications, and/or diabetes knowledge through patient education. Tailoring is defined as any education, feedback, or communication provided which is based on the interest, physiological, and behavioral condition of the participating individuals [25].

### Type of comparators

Non-eHealth interventions or usual care will be considered as comparators.

### Outcome measures

The main outcome of interest in this review is the mean difference in glycated hemoglobin (HbA1c) levels pre-/post-intervention.

### Information sources

An intensive search in a set of databases will be conducted by the first author. Searches will be performed in PubMed, ISI Web of Science (Science Citation Index), and PsycINFO. We will search peer reviewed journal articles published in English and German.

### Search strategy

Relevant keywords that were identified during scoping, such as “type 2 diabetes,” “diabetes type 2,” “eHealth,” “telemedicine,” “telehealth,” “mHealth,” mobile health, “web-based” “Internet,” “digital media,” “short message service,” “text message,” “videogame,” and “health game” will be used as keywords. In the PubMed search, the phrase “Title/Abstract” will be connected with the keywords to look for relevant articles based on their titles and abstracts. The Boolean operators “AND” and “OR” will be used to connect the keywords. Then the full texts of the peer reviewed articles meeting our inclusion criteria will be retrieved. The PubMed search strategy is included in the supplementary material of this protocol (see Additional file 2). The search strategy will be modified to meet the requirements of the other databases.

### Data management

All bibliographic data of our search results will be transferred to EndNote X7 reference Information System software [26] so that duplicate records can be removed. The online screening tool Covidence will be used to import the set of de-duplicated citations and to manage the title and abstract screening process.

### Selection process

Two reviewers (MK and CP) will independently screen the titles and abstracts of the articles identified in the electronic search. Articles which do not satisfy the eligibility criteria will be removed. Full texts of the remaining articles will be retrieved and screened, according to the eligibility criteria, to identify articles to be included in the final review. Reasons for exclusion of any articles will be recorded. Bibliographies of the selected articles will be hand searched to identify any relevant studies that might not have been included. Results of both authors will be compared in the presence of a third author. In case of a disagreement, the decision of a third reviewer, one of the co-authors, will be considered final.

### Quality assessment

To assess the quality of studies, the Cochrane Collaboration’s tool for assessing risk of bias in randomized trials will be used [27]. Studies will be evaluated against the predefined criteria. Sequence generation, allocation concealment, blinding of participants, personnel and outcome assessors, incompleteness of outcome data, selective outcome reporting, and other sources of bias from each study will be assessed.

### Extraction of data

An Excel data extraction form will be prepared by MK (see Additional file 3). Data from studies will then be extracted by MK and CP using the extraction format. Discussion will be held in case of disagreements.

### Data items

Information on the salient features of the included studies, i.e., title, study setting (country, study population), year of publication, sample size, type of intervention/s, outcome/s, duration of follow-up, intervention period, intervention effect, HbA1c changes (and *p* values), confidence intervals, and the type of statistical analysis will be extracted from the included articles. In addition, BCTs employed in the interventions will be identified based on the description of interventions provided in the articles. The BCTs of the interventions will be independently be coded by two reviewers (i.e., two of the authors of this protocol) who have experience with using the most recent and comprehensive BCT taxonomy [19]. In the event of any disagreements between two authors at any stage of the review process (i.e., during the screening of titles and abstracts, quality assessment, data extraction, coding using the BCT taxonomy), a third author will be consulted, whose decision will be final.

### Outcome prioritization

The main outcome of interest of this review is the mean difference in glycated hemoglobin (HbA1c) percentage pre-/post-intervention and between intervention and control or usual care groups.

### Strategy for assessing the risk of bias in the individual studies

The risk of bias in the included studies will be assessed by three reviewers (TLH, ZK, and LC), using the Cochrane risk of bias tools for RCTs [27]. Based on this assessment tool, studies will be rated as having a low, high or unclear risk of bias. If two authors have disagreements, a third author will be consulted for resolving the discrepancy to reach consensus.

### Missing data handling mechanisms

Whenever there is a lack of information necessary for this review, the authors will contact the corresponding author of the respective article to request the relevant data via email or phone calls. If there is no response after two attempts to contact the corresponding author (within a maximum of 1 month), the article will be excluded from the quantitative synthesis. The reason for the exclusion, along with the citation of the individual article will be provided. However, the authors of the review may still decide to include the article in the narrative synthesis, should there be relevant information included in the article.

### Assessment of heterogeneity and data synthesis

Cochrane collaboration Review Manager Version 5.3 will be used to analyze the data [28]. The difference in effectiveness of type and number of BCTs on HbA1c will be analyzed using Stata version 14. The effect sizes of individual BCTs will be calculated in a moderator analysis using the meta-regression model or random effect model. Meta-analysis will only be performed if there is sufficient homogeneity in outcomes between at least two studies. For meta-analysis, the statistical heterogeneity will be computed using the I-squared statistic and evaluated against the Cochrane’s chi-square test using a 10% significance level. Pooled effect size estimates with a 95% confidence interval that measure the size of the intervention effect across the studies will be calculated. If the heterogeneity remains significant and meta-analysis is not feasible, a narrative synthesis will be carried out using the framework by Popay and colleagues [29]. The narrative synthesis of all relevant studies will include tables of the study and participants’ characteristics, intervention components, settings that interventions were implemented in, and mean change in HbA1c in both control and intervention groups.

### Sensitivity analysis

Sensitivity analysis based on study design and study quality (i.e., studies having a low risk of bias will be compared to studies having a high risk of bias based on the Cochrane risk of bias tool) will be performed to determine the robustness of the results. We will also perform sensitivity analysis to evaluate whether the choice of the statistical model (random effect vs. fixed effect) affects the results.

### Subgroup analysis

To determine differential effects on HbA1c, subgroup analyses by type (tailored vs. untailored, theory vs. not theory-based) and duration of intervention, and mode of delivery (apps, games, web-based, computer, and mobile phone) will be performed.

### Meta-bias

The risk of bias will be assessed using the Cochrane tool for assessing risk of bias [27]. The risk of meta-bias will be assessed using funnel plots. Whenever there is no or only minimal risk of bias, the studies will be symmetrically distributed about the mean effect size. Whenever there is a higher risk of bias, there will be symmetry at the top of the funnel plot, a few studies in the middle and more studies at the bottom of the funnel plot will be missing [30].

### Confidence in cumulative evidence

The quality of the synthesized evidence will be evaluated using GRADE (Grading of the Recommendations Assessment, Development and Evaluation) [31, 32]. The quality of evidence will be ranked as very low, low, moderate, and high. Two reviewers (MK and CP) will independently evaluate the quality of evidence utilizing the GRADE profiler (GRADEpro) software [32]. Subsequently, data will be imported from RevMan 5, and GRADEpro will be used to tabulate summary findings.

## Discussion

Behavior change interventions help persons with T2DM improve self-management behaviors, adhere to medications, follow specific dietary recommendations, and increase physical activity [33–36]. Due to its comprehensiveness and reliability, an increasing number of systematic reviews are employing the BCT taxonomy to synthesize evidence regarding active ingredients of behavioral interventions [19].

Currently, there is a lack of reviews aimed at assessing the effectiveness of individual BCTs employed in eHealth interventions aimed at improving glycemic control among persons with poorly controlled T2DM. This review will be one of the first to shed further light on the question which BCTs are essential in eHealth interventions designed to improve glycemic control. Furthermore, results of this review may inform the development of future interventions targeting this population.

### Presentation and reporting of the results

This systematic review protocol will follow the recommendations of the Preferred Reporting Items for Systematic Review and Meta-Analysis Protocols 2015 Statement (PRISMA-P) [24].

## Additional files

Additional file 1: PRISMA-P 2015 checklist (DOC 100 kb)

Additional file 2: PubMed search strategy (DOC 34 kb)

Additional file 3: Data extraction template. (XLS 34 kb)

## Abbreviations

BCT: Behavioral Change Techniques
GRADE: Grading of the Recommendations Assessment, Development and Evaluation
HbA1c: Glycated hemoglobin
PRISMA: Preferred Reporting Items for Systematic Review and Meta-Analysis
RCT: Randomized Controlled Trial
T2DM: Type 2 diabetes mellitus
