# Supplementary material for: Effectiveness of behavioral change techniques employed in eHealth interventions designed to improve glycemic control in persons with poorly controlled type 2 diabetes: a systematic review and meta-analysis protocol
Source: Syst Rev. 2017 Oct 24;6:211. doi: 10.1186/s13643-017-0609-1 (PMC5655972; doi:10.1186/s13643-017-0609-1)
Supplement: Supplementary file 2 — PubMed search strategy (DOC 34 kb) [file 13643_2017_609_MOESM2_ESM.doc]

Additional file 2: PubMed search Strategy

| **Search** | **PubMed** |
| --- | --- |
| **#1** | eHealth[Title/Abstract] |
| **#2** | Telemedicine[Title/Abstract] |
| **#3** | Telehealth[Title/Abstract] |
| **#4** | mHealth [Title/Abstract] |
| **#5** | mobile health[Title/Abstract] |
| **#7** | *web based* |
| **#8** | *internet[Title/Abstract])* |
| **#9** | SMS[Title/Abstract] |
| **#10** | *short message service[Title/Abstract]* |
| **#11** | *text message[Title/Abstract]* |
| **#12** | *videogame[Title/Abstract]* |
| **#13** | *Health game[Title/Abstract]* |
| **#14** | Type 2 diabetes [MeSH] |
| **#15** | #1 OR #2 OR #3 OR #4 OR #5 OR #6 OR #7 OR #8 OR #9 OR #10 OR #11 # OR #12 OR #13 |
| **#16** | #14 AND #15 |
